# Deep Brain Stimulation for Obesity

**DOI:** 10.7759/cureus.259

**Published:** 2015-03-25

**Authors:** Allen L Ho, Eric S Sussman, Michael Zhang, Arjun V Pendharkar, Dan E Azagury, Cara Bohon, Casey H Halpern

**Affiliations:** 1 Department of Neurosurgery, Stanford University School of Medicine; 2 Department of Neurosurgery, Stanford School of Medicine/Stanford University Medical Center; 3 Department of Neurosurgery, Stanford University Medical Center; 4 Department of Surgery, Stanford School of Medicine/Stanford University Medical Center; 5 Department of Psychiatry and Behavioral Sciences, Stanford University School of Medicine

**Keywords:** deep brain stimulation, obesity, hypothalamus, lateral hypothalamus, nucleus accumbens, metabolism, reward pathway, neuromodulation, food, behavior

## Abstract

Obesity is now the third leading cause of preventable death in the US, accounting for 216,000 deaths annually and nearly 100 billion dollars in health care costs. Despite advancements in bariatric surgery, substantial weight regain and recurrence of the associated metabolic syndrome still occurs in almost 20-35% of patients over the long-term, necessitating the development of novel therapies. Our continually expanding knowledge of the neuroanatomic and neuropsychiatric underpinnings of obesity has led to increased interest in neuromodulation as a new treatment for obesity refractory to current medical, behavioral, and surgical therapies. Recent clinical trials of deep brain stimulation (DBS) in chronic cluster headache, Alzheimer’s disease, and depression and obsessive-compulsive disorder have demonstrated the safety and efficacy of targeting the hypothalamus and reward circuitry of the brain with electrical stimulation, and thus provide the basis for a neuromodulatory approach to treatment-refractory obesity. In this study, we review the literature implicating these targets for DBS in the neural circuitry of obesity. We will also briefly review ethical considerations for such an intervention, and discuss genetic secondary-obesity syndromes that may also benefit from DBS. In short, we hope to provide the scientific foundation to justify trials of DBS for the treatment of obesity targeting these specific regions of the brain.

## Introduction and background

Obesity is one of the most pressing public health issues in the United States. Obesity increases the risk of cardiovascular disease, diabetes, and cancer and is associated with a diminished quality of life and up to a 20-year decrease in life expectancy [1-4]. Currently, more than two-thirds of adult Americans are overweight and over one-third are obese [5-6]. Obesity is now the third leading cause of preventable death in the US, accounting for 216,000 deaths annually and nearly 100 billion dollars in health care costs [7-8]. Unfortunately, conservative measures are associated with high rates of relapse, and thus surgical treatment options have gained favor [9-11]. Advancements in bariatric surgery have allowed for significant weight loss in > 90% of patients [12]. Interestingly, in addition to the anatomic sequelae of obesity surgery, which impose mechanical limitations on the magnitude of food consumption, neuroendocrinological effects appear to play a significant role in the efficacy of such procedures. Several studies have demonstrated postoperative changes in levels of circulating gut peptides that project to the brain [13], thereby underscoring the critical importance of central nervous system’s feeding and satiety centers in the pathogenesis of obesity. Unfortunately, however, substantial weight regain and recurrence of the associated metabolic syndrome still occurs in almost 40% of patients over the long-term, necessitating the development of novel therapies [14-15].

Our continually expanding knowledge of the neuroanatomic and neuropsychiatric underpinnings of obesity has led to increased interest in neuromodulation, similar to other treatment-refractory disorders, such as obsessive-compulsive disorder. Deep brain stimulation (DBS) provides reversible electrical stimulation of neural circuitry and has been utilized as an effective and safe therapy for a wide variety of neurologic disorders [16-18]. While the precise mechanism of DBS remains unclear, it is well-established that high-frequency electrical stimulation clinically mimics the effects of neural ablative procedures [19-20]. However, the ability to titrate and/or reverse the effects of DBS make it the preferred method of neuromodulation [21-24]. Recent clinical trials of DBS in chronic cluster headache, Alzheimer’s disease, and depression and obsessive-compulsive disorder have demonstrated the safety and efficacy of targeting the hypothalamus and reward circuitry of the brain with electrical stimulation, and thus provide the basis for a neuromodulatory approach to treatment-refractory obesity [16, 25-27].

While the role of the hypothalamus in the neurophysiology of obesity has been well-established for decades [28-29], more recent investigation has verified the importance of the brain’s reward circuitry in the pathologic food-seeking behaviors typically seen in obesity [30-32]. This finding makes the nucleus accumbens (NAc) another favorable target for neuromodulation. In this study, we review the literature implicating these targets for DBS in the neural circuitry of obesity. We will also briefly review ethical considerations for such an intervention and discuss genetic secondary-obesity syndromes that may also benefit from DBS. In short, we hope to provide the scientific foundation to justify trials of DBS for the treatment of obesity targeting these specific regions of the brain.

## Review

### Lateral hypothalamus as a target for DBS

**Figure 1:**
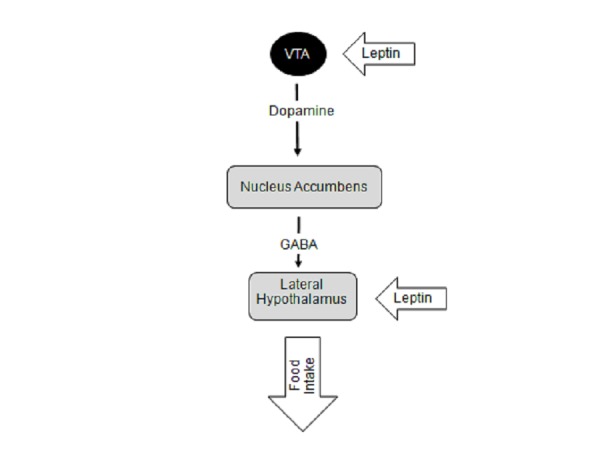
Schematic diagram depicting the deep brain stimulation (DBS) targets for obesity and their role in homeostatic pathway of energy balance The LH is responsible for providing anabolic feedback onto the autonomic nervous system effectors. The nucleus accumbens (NAc) is the center of the reward pathway in the brain integrating inputs from various high cortical brain areas and the limbic system to reinforce certain beneficial behaviors, such as feeding. Integration of the reward pathways with feeding behavior begins with dopamine release from the ventral tegmental area (VTA) neurons that project onto the nucleus accumbens (NAc). Within the NAc, there are neurons that projection onto the lateral hypothalamus (LH) which contain neurons that stimulate food intake. These nuclei also respond to various hormonal peptides, such as leptin, that are released by the the metabolic systems of the body that link food intake and energy metabolism to the reward pathways within the brain.

The hypothalamus is divided into multiple distinct functional regions; the main subregion that has received the most focus as a target for DBS is the lateral hypothalamus (LH). The LH has classically been recognized as the feeding center, providing anabolic control over the body's metabolism (Figure 1) [25]. The LH contains neurons that produce two orexinergic neuropeptides known as orexin and melanin-concentrating hormone (MHC). Intracerebroventricular infusion of either peptide elicits feeding [33]. Orexin-containing neurons project to various brain areas regulating feeding behavior. Over-expression of MCH in experimental models of obesity has been associated with insulin resistance and obesity, whereas MCH-knockout mice tend to be hypophagic and lean [34]. A variety of other peptides in addition to orexins have been implicated in LH activity, such as neuropeptide Y^68^, and agouti-related protein [35-39]. Moreover, the LH is one of the main regions within the hypothalamus that expresses the leptin receptor. Indeed, the activity of these orexin-containing neurons is mitigated by the presence of leptin, as endogenous leptin signaling in the hypothalamus restrains the overconsumption of calorically dense foods [40]. Animal studies and human genetic studies have confirmed that leptin deficiency is associated with a predisposition to obesity [41-43]. Whether by an inability of leptin to reach its neural target, a decrease in leptin isoforms, or decreased expression of leptin receptor [44-45], this “leptin resistance” lends further evidence that the LH is dysregulated, leading to the hypothesis that targeting this region with DBS may disrupt this aberrant circuitry and ameliorate the obese state [46-48]. 

In support of this hypothesis, early lesion studies in rats induced leanness, suggesting an important role of the LH in exerting an anabolic effect on the body's metabolic systems [49-50] (Figure 1). Functionally impairing the endogenous activity of the LH with DBS is thought to mimic the effects of these lesions. This stems from experiences with subthalamic nucleus DBS for Parkinson disease, whereby chronic stimulation has the same clinical effects on parkinsonian features as subthalamotomy [51]. Indeed, bilateral DBS studies in rats have demonstrated a 16% weight loss [52]. Meanwhile, in a recent pilot study of LH DBS in humans, three morbidly obese patients who had previously failed to respond to gastric bypass surgery demonstrated an increase in resting metabolism at their three-year follow-up. Notably, extended follow-up demonstrated a sustained increase in resting metabolic rate with some weight loss in two out of three patients, and without any significant detrimental psychological consequences [53]. A key observation from this early work is the size of the region that DBS must modulate. An increase in metabolism can be achieved with a region as small as 2 mm^2 ^despite the LH’s anatomical size measuring approximately 6 x 5 x 3.5 mm laterally, anteroposteriorly, and dorsoventrally, respectively [54]. These studies have identified the LH as a promising target for DBS for obesity, however, future clinical studies must verify the optimal location for LH DBS.

### Nucleus accumbens and the reward pathway

Because it houses the hunger and satiety centers of the brain, the hypothalamus has traditionally been the focus of obesity neuromodulation, as detailed above [28-29]. However, many individuals with obesity exhibit many behavioral features of addiction-like behavior, such as binge eating, that is known to be related to dysfunctional reward circuitry in the brain [30-31, 55]. Feelings of craving, reward anticipation, consumption driven reward, and withdrawal are all modulated by the mesocorticolimbic dopaminergic circuitry, which converges on the nucleus accumbens (NAc) [56-57]. Anatomically, the reward circuitry of the brain is composed of dopamine-secreting ventral tegmentum neurons that project to the NAc via the medial forebrain bundle (Figure 1) [58-60]. Access to such a highly palatable, high-caloric diet in rodents has been shown to heighten dopaminergic activity in the brain, which reinforces binge-eating behavior [61-62]. Multiple animal studies of chronic exposure to high-fat diets have demonstrated similar alterations in food consumption mediated by loss of both inhibitory control and withdrawal symptoms[63-64]. Mice conditioned to a high fat diet continuously endure harsh environments to maintain this palatable diet and demonstrate evidence of physiologic withdrawal when weaned from it [66]. A significant increase in markers of stress and decreased dopaminergic signaling within the NAc is seen in these animals after withdrawal from high fat diets [62].

In addition, rodent studies have demonstrated the biochemical, neuroendocrinological, neuroanatomical, and behavioral connections between the lateral hypothalamus and NAc (Figure 1) [65-66]. It is established that glutamate neurons in LH are a major projection site of NAc output neurons, and the NAc is the only striatal region that sends projections to LH [67]. The leptin signaling pathway as well as other gut-derived neuropeptides, such as hormone peptide YY, glucagon-like-peptide 1, and ghrelin, have also been found to project onto this circuitry (Figure 1) [46, 48, 68]. Ventral tegmental dopamine neurons express the leptin receptor and respond to leptin with a reduction in firing rate [68]. Direct administration of leptin to this midbrain structure caused decreased food intake while long-term knockdown of the leptin receptor led to increased food intake, locomotor activity, and sensitivity to highly palatable food. These data support a critical role for the leptin signaling pathway that involves this reward circuitry and the hypothalamic area in regulating feeding behavior. Moreover, this provides functional evidence for direct action of a peripheral metabolic signal on the reward circuit.

In humans, functional imaging studies have played a critical role in establishing the important involvement of the NAc in behaviors associated with obesity. fMRI studies of patients imagining intake of palatable foods found altered activation in the ventral striatum in individuals more at risk for future weight gain [69]. fMRI studies of response to images of high (vs. low) calorie foods or to anticipated receipt of a sweet taste in obese (vs. lean) participants found greater activity in the NAc [70-71]. Decreases in response of the NAc to high (vs. low) calorie food images were found in patients one-month post-roux-en-Y gastric bypass surgery [72]. Two preliminary studies also found a trend for altered dopamine receptor binding potential in the ventral striatum postoperatively in five patients [73-74]. Thus, the NAc must be considered as a potential target for neuromodulation of reward circuitry to control the behavioral patterns of food dependence seen in obese individuals [75].

### Nucleus accumbens as a target for DBS

Classically, the anatomical posterior border of the NAc has been at the level where the anterior commissure becomes discontinuous, and it is this posterior region of the nucleus that has achieved the best results when targeted for DBS for psychiatric disorders [76-77]. Proof-of-concept lesionectomy studies in rodent models have supported the potential efficacy of targeting the NAc with DBS for obesity. For example, in rats given stereotactic 6-OHDA infusions into the NAc, food hoarding behavior was virtually eliminated, and these animals experienced significant weight loss. These effects were readily reversed with levodopa administration [78]. DBS stimulation of the NAc has been performed in multiple animal studies with inconsistent results with regard to effects on feeding behavior, but most studies have not specifically examined the effects of DBS on animal models of obesity or eating disorders [79-81]. A recent study by Halpern, et al. demonstrated that DBS of the anteromedial NAc, but not the dorsal striatum, led to a decrease in binge-eating behavior, and this effect was mediated by dopamine signaling involving D2 receptors. This underscores the specificity of involvement of the mesolimbic pathways in food intake related reward pathways. The authors also examined the effects of chronic DBS of the NAc in diet-induced obese mice, and found that NAc stimulation led to decreased caloric intake, sustained weight loss, and improvements in features of Type 2 diabetes [82]. The NAc is a well-validated DBS target, and the safety and efficacy of this anatomic target in humans have already been demonstrated for treating disease processes, such as treatment-refractory depression, OCD, and alcoholism [83-85]. Thus, given the role of the NAc in food-seeking behavior, the NAc is a suitable target for a clinical trial for DBS treatment of refractory obesity.

### Ethical considerations

Data from animal research [52, 86-87] as well as from recent case reports and pilot studies in humans [84, 88] have demonstrated the potential of obesity to be therapeutically targeted via DBS. Given the significant data accumulated from animal studies and case reports of obesity treatment with DBS, the specter of a clinical trial of DBS for obesity has raised some ethical considerations. There is significant overlap between obesity and addiction, raising concern for maladaptive behavior as a result of imperfectly executed neural manipulation of the CNS reward circuitry [89]. As with any new treatment for addictive behavior, the possibility for threatened autonomy in the face of behavior-altering treatment is often discussed [90]. Decision-making autonomy prior to treatment is typically preserved in obese patients without other psychiatric or developmental abnormalities as long as informed consent is obtained [91]. Reports of altered behavior ranging from emotional hyperactivity to increased impulsivity to suicidality have been reported [92-93], demonstrating that threatened autonomy can occur in the context of treatment. However, four basic demands for autonomous action include the ability to understand, appreciate, evaluate, and control one's actions in the context of treatment [91]. DBS for morbidly obese patients general satisfy the first three of these requirements, and ultimately goal of treatment would be to attain the fourth in terms of self-control over food consumption.

Given this, we firmly believe that the medical need and scientific justification for treatment of metabolic and eating disorders associated with obesity greatly outweigh the theoretical ethical risks as long as the treatment population is carefully selected. That is, a trial of DBS in obesity should be restricted to treatment refractory patients who have been cautiously evaluated by a multidisciplinary team of obesity specialists, ethicists, and neurosurgeons, and deemed medically and psychologically prepared for postoperative management. Stimulatory parameters of the hypothalamus and reward circuitry should also be carefully studied and modulated on an individual basis so as to not detrimentally alter metabolism or the reward circuitry. Targeting the NAc, in particular, could decrease the reinforcing sensation of consumption, serve as a substitute for the reward of eating, attenuate craving, inhibit a sense of withdrawal, or any combination of these effects. The question of whether attenuation of the reward sensation of food consumption could be achieved without altering a patient’s ability to experience other normal pleasure remains to be answered. In light of these considerations, extensive study is necessary to define the parameters of stimulation for optimal safety and efficacy. While animals studies will be crucial for defining neural targets, stimulatory parameters, laterality, and mechanism of DBS for obesity, the translation of animal models to human study is not always seamless, and human study will likely have to occur in parallel [94].

### Secondary obesity syndromes

*Prader-Willi Syndrome* (PWS) is characterized by extreme hyperphagia, obesity, and intellectual disability, and is caused by a genetic defect resulting in absent expression of several imprinted genes in the 15q11-q13 region from the paternal chromosome 15 [95]. PWS patients are often morbidly obese due to their insatiable appetites [96]. One out of three PWS patients are over 200% ideal body weight, and there have been reports of overconsumption leading to stomach rupture in these patients [97]. The metabolic profile of PWS includes increased adipose to lean mass ratio [98-99], decreased total and resting energy expenditure [100], and elevated fasting ghrelin levels [101]. Despite the most radical medical and surgical interventions, PWS remains difficult to treat. In particular, bariatric surgery has had limited effectiveness and a concerning safety profile, given the increased medical comorbidity in this population [102].

PWS individuals likely have a disruption of basic satiety mechanisms leading them to consume more and for longer periods of time than obese individuals [103-104]. These disruptions manifest as post-meal hyperactivation of the hypothalamus, NAc, amygdala, hippocampus, medial prefrontal cortex (PFC), OFC, and insula, regions involved in both the food satiety and reward circuitry [105-108]. fMRI studies have demonstrated that prior to consumption, individuals with PWS exhibit higher activity in reward/limbic regions (NAc, amygdala) and lower activity in subcortical hunger and satiety regions (hypothalamus, hippocampus), but post-consumption exhibit high activity in subcortical regions and lower activity in inhibitory pre-frontal cortical regions (posterior/lateral OFC, DLPFC) compared to controls. Thus, PWS not only leads to greater activation of reward and hunger centers in anticipation of food, but also disrupts inhibitory circuitry post-prandially [109].

PWS is a common link between food consumption and reward pathways in the brain that when disturbed leads to uncontrolled feeding and morbid obesity (Figure 1). As mentioned previously, LH and NAc are potential targets for PWS that have already been targeted in other disease states. Specifically, the LH has already been targeted via DBS for obesity [53-54] and headache [110] and the NAc for OCD, anxiety, addiction, and depression [76-77]. We propose that these same targets may be potential targets for DBS for PWS.

*Kleine-Levin Syndrome* (KLS) is a rare episodic hypersomnia disorder that is also characterized by hyperphagia, as well as hypersexuality and cognitive impairment [111-113]. Between episodes, clinical symptoms may resolve entirely. While the pathophysiology of this disorder is largely unknown [113], recent imaging studies have identified aberrations in a variety of deep brain nuclei and cerebral cortical areas. 

A 2014 functional MRI (fMRI) study of KLS patients during acute episodes identified hyperactivation of the left thalamus, as well as hypoactivation of the anterior cingulate cortex and medial prefrontal cortex [114]. Another recent study comparing brain perfusion scintigraphy in KLS patients and healthy controls identified hypoperfusion in the hypothalamus, thalamus, caudate nucleus, and frontal and temporal cortical associative areas [113, 115]. While significant progress remains to be made with regard to the pathophysiology underlying this disease, the identification of abnormalities in several deep brain structures raises the possibility of targeted DBS treatment for KLS.

## Conclusions

In this study, we reviewed the literature implicating the lateral hypothalamus and nucleus accumbens, well-validated DBS targets, in the neural circuitry of obesity. We also presented the current data supporting DBS targeting of these foci to modulate both the metabolic and behavioral pathophysiology involved in treatment-refractory obesity, briefly reviewed ethical considerations for such an intervention, and discussed genetic secondary-obesity syndromes that may benefit from DBS. Though there have been no human studies to date specifically utilizing DBS towards the treatment of obesity, we have provided the scientific foundation and justification for a DBS trial for the treatment of obesity targeting these specific regions of the brain.
